# Prospective, randomized, single blinded pilot study of a new FlatWire based sternal closure system

**DOI:** 10.1186/1749-8090-9-97

**Published:** 2014-06-02

**Authors:** Ashley N Boustany, Paul Ghareeb, Kee Lee

**Affiliations:** 1 Department of Surgery, Division of Plastic Surgery, University of Kentucky, Lexington, USA; 2 Department of Surgery, Division of Plastic Surgery, Emory University, Atlanta, USA; 3 Monongalia General Hospital, Thoracic and Cardiovascular Surgery, Morgantown, WV, USA; 4 Division of Plastic Surgery, University of Kentucky, 138 Leader Avenue, Lexington, KY 40506-9983, USA

**Keywords:** Sternal closure, Figure 8 FlatWire, Sternal stability, Sternal pain, Sternal dehiscence, Median sternotomy

## Abstract

**Background:**

Unstable steel wire cerclage following open heart surgery may result in increased pain, sternal cut-through, non-union, or dehiscence. These complications lead to longer hospital stays, increased cost, higher morbidity, and patient dissatisfaction. The Figure 8 FlatWire Sternal Closure System is a new construct which is a simple, intuitive, and inexpensive alternative for primary sternal repair following open heart surgery. Prior bench-top testing of FlatWire has demonstrated superior strength and stiffness compared to traditional steel wire. We present our initial experience in a prospective, randomized, single blinded pilot study utilizing this FDA approved system.

**Methods:**

Sixty-three patients undergoing elective complete sternotomies at a single institution were randomly assigned to receive either the Figure 8 FlatWire or standard steel wire cerclage. All surgeries were performed by a single board certified cardiothoracic surgeon. Data collected included: Age, BMI, pump time, off pump to surgical stop time, length of hospital stay after surgery, cost from time of surgery to discharge, and pain on a visual analog pain scale on the day of discharge, day 30, and day 60.

**Results:**

The groups were well matched. Patients receiving the Figure 8 FlatWire (33) had a reduction in length of stay compared to patients receiving steel wire circlage (30), but it was not statistically significant (6.8 vs. 7.8 days respectively, p < 0.093). Additionally those with the FlatWire reported significantly decreased pain at day of discharge (3.07 vs. 4.92 points on pain scale, p < 0.0066), with similar pain scores at 30 and 60 days. Off pump to surgery stop time was increased by 15.9 minutes in patients receiving the FlatWire vs. steel wires (55.7 vs. 71.6 minutes, p = 0.00025). Mean cost from surgery until discharge was $87,820.98 in the FlatWire group vs. $91,930.29 in the steel wire group (p < 0.3082).

**Conclusion:**

Early clinical results suggest that Figure 8 FlatWire provides excellent stability, which resulted in significantly diminished postoperative pain at discharge. Although not significant there was a trend toward decreased length of stay, and reduced cost. Further clinical research is warranted to expand upon these initial trends and validate long term outcomes.

## Background

Closure of the median sternotomy with steel wire has been used for more than 50 years [1]. However, the patient population undergoing cardiac surgery today has dramatically changed over the last 20 years. Surgical candidates today present as older, multi-morbid patients, with more serious cardiac disease. Advanced age, diabetes, obesity, renal insufficiency, lung disease, osteoporosis, and poor nutritional status compounded by more complex operations are commonplace [1].

The consequences of sternal separation, cut-through, or dehiscence can be profound, with a mortality rate of 10-40% [2]. The risk of complications increase in complex patients and in the elderly [3,4]. Over 760,000 procedures requiring sternotomy are performed every year, making complications a serious healthcare issue [5,6].

The median hospital costs for patients developing sternal wound complications following coronary bypass grafting (CABG) can be up to 2.8 times higher than for uncomplicated patients [7]. The Department of Health and Human Resources has identified these complications as hospital-acquired events for which hospitals should not receive additional payment if the condition was not present upon admission.

The most important factor in the prevention of sternal events is a stable sternal approximation following sternotomy [8,9]. There have been many attempts to improve upon the standard method of steel wire cerclage, but these systems have failed to gain widespread adoption due to practicality or excessive cost.

The Figure 8 FlatWire Sternal Closure System was developed to provide a primary closure method that is superior to standard steel wire cerclage, while avoiding the drawbacks of current wire alternatives. Prior bench-top comparison demonstrated that FlatWire is 70% stronger than standard wire when placed in a transverse configuration, and 40% stronger when used in the crossed (“X”) pattern. In addition, the device demonstrated significantly reduced cut-through and improved lateral and longitudinal cyclical testing when compared to steel wire cerclage [7].

The authors hypothesized that the superior mechanical aspects of FlatWire would translate into improved clinical outcomes by reducing post-operative pain, leading to decreased length of stay and total cost.

### Technology and technique

**Figure 1:**
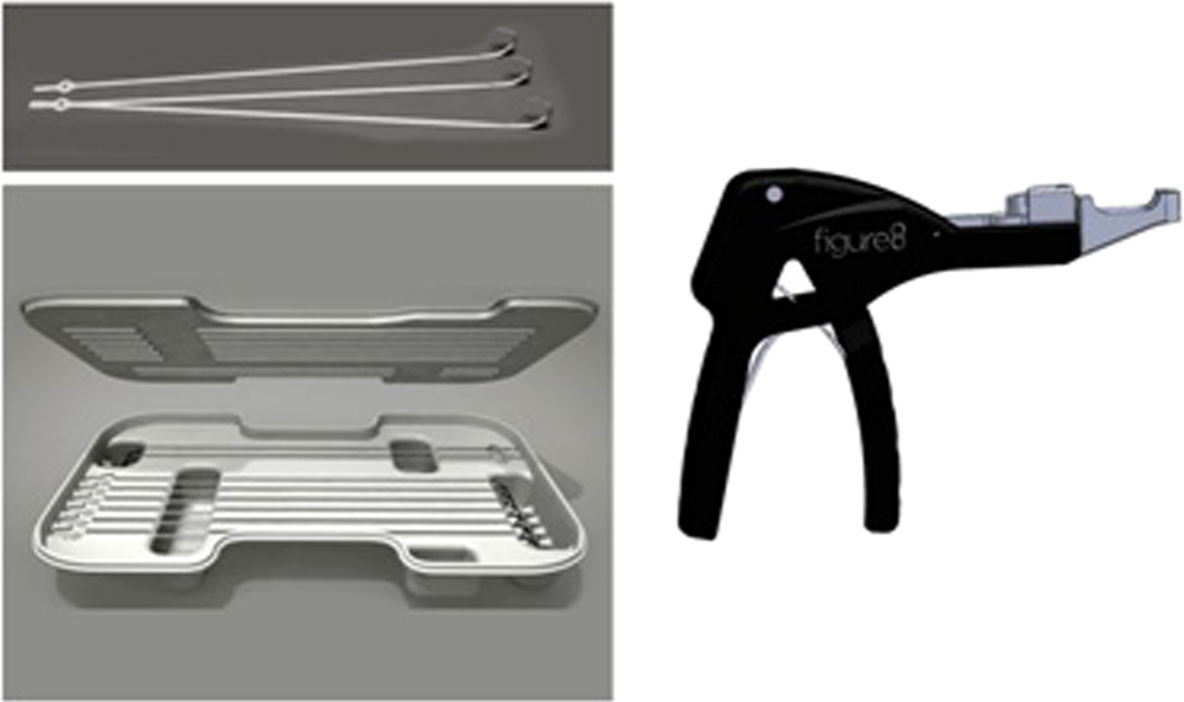
**FlatWire sternal closure system.** The Figure 8 FlatWire Sternal Closure System comes packaged with 6 straight and 2 X designed FlatWires. A reusable tensioning device is provided to tighten the straps and re-approximate.

The Figure 8 FlatWire is made of 316 L stainless steel. A stainless steel needle is attached to the flexible body of the FlatWire. The body sequentially widens to 2.8 mm which facilitates delivery around the sternum. It comes packaged as a complete surgical kit with eight FlatWires and a reusable aluminum tensioning device (Figure 1).

A standard needle driver and wire cutters are also used for application. The FlatWires have two distinct ends: a proximal end attached to a needle and a distal end with a rotating central hub and laser welded security box that can withstand >1400 N of force.

**Figure 2:**
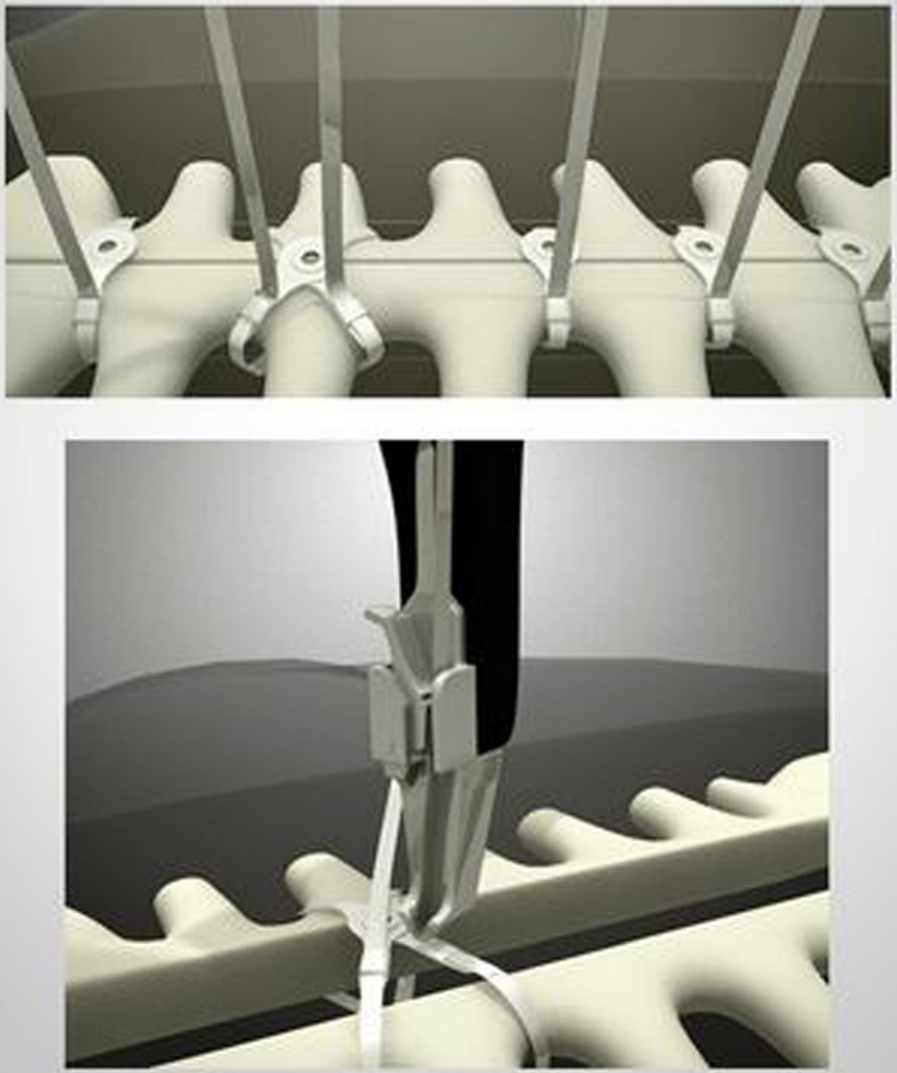
**FlatWire configurations.** FlatWires placed in the straight or figure 8 position are placed as described, tightened with the tensioning device, and then cut with standard wire cutters.

A needle driver is used to feed the FlatWire around the sternum while its supported in its central arc to minimize kinking. The needle is removed with standard wire cutters and the FlatWire end is fed through the security box. It is temporarily secured and left in position until remaining straps are placed, working in a cephalad to caudad manner (Figure 2, video: https://www.youtube.com/watch?v=h0NcrmR5NDc).

Once the desired number and configuration of FlatWires are placed, the Figure 8 tensioning instrument is used approximate the sternal halves. The tensioning instrument has a custom built in break away mechanism that prevents applied forces to exceed 300 N. FlatWires are then tensioned to the desired effect using tactile feedback and visual cues. The strap is temporarily bent to 90 degrees to hold the sternal position. At this point the closure is reversible by unbending the FlatWire. Once the surgeon is satisfied with sternal approximation; the FlatWires are simply twisted 120 degrees, without tension, using the instrument and cut to 1 cm. The 1 cm end is folded in half and down onto the sternum. FlatWires may be used in conjunction with or as a replacement for standard steel wire in primary sternal closure. If emergent thoracic re-enetry is necessary, FlatWire removal is simple and fast using standard steel wire cutters; the same wire cutters that are used to remove standard steel sternotomy wires.

## Methods

**Figure 3:**
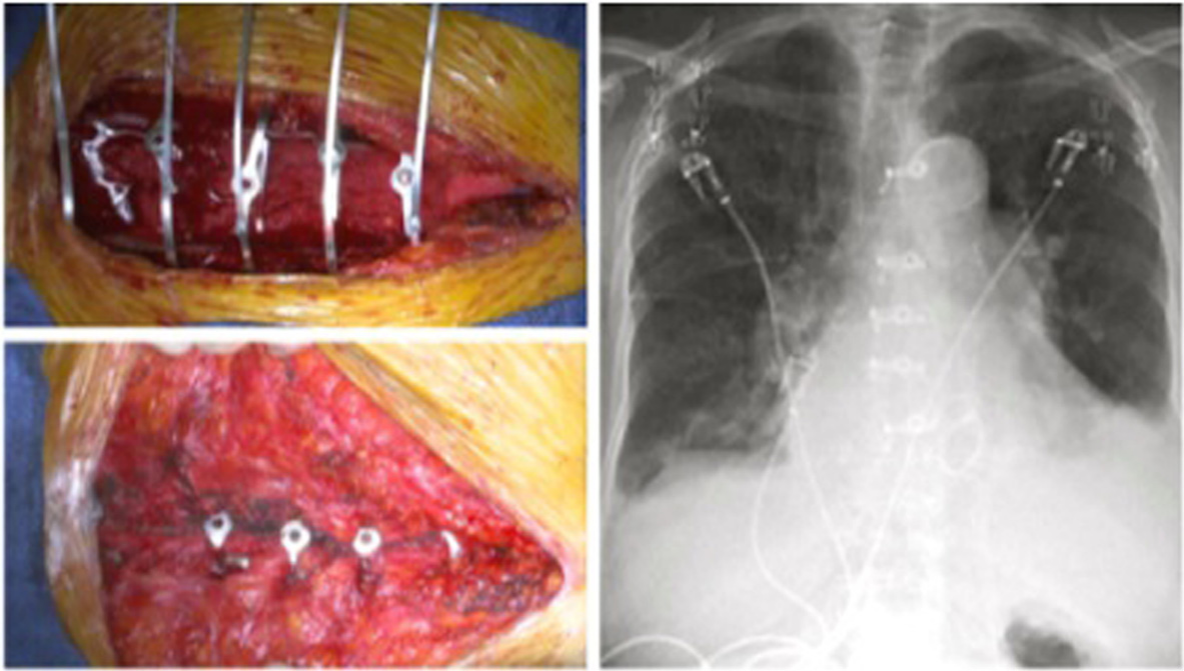
**Intra-operative placement and post-operative X-rays.** FlatWires are trimmed after all are placed, allowing for adjustments to be made. X-rays at day of discharge and 30 days confirmed alignment and proper placement of sternal constructs.

Monongalia Institutional Review Board approved the research protocol. Sixty-three patients scheduled to undergo elective complete sternotomy at a single institution were randomly divided into two cohorts: one group received the Figure 8 Flatwires while the other received standard steel wire cerclage (No. 7 gauge stainless steel wire, Ethicon Ltd.). The patients were blinded as to which group they were in. The same board-certified cardiothoracic surgeon performed each procedure, and the configuration of the closure method was determined by surgeon’s clinical judgment. X-rays at day of discharge and 30 days confirmed alignment and placement (Figure 3).

Pain was measured on a 10 point visual analog pain scale at day of discharge, day 30, and day 60. Length of postoperative stay was recorded and total cost from surgery to discharge was calculated. The time of surgical closure was collected as the cardiopulmonary bypass pump off time to the surgery end time (recorded by anesthesia). Complications were documented.

Statistical analyses were performed using SAS software (Version 9.2; Cary, NC); a student t-test was utilized to compare pain scores and length of stay between the two groups.

## Results

A total of 63 patients underwent coronary artery bypass grafting with midline sternotomy, 30 patients received traditional stainless steel wire sternal cerclage and 33 patients received FlatWires. Mean patient age, body mass index, and pump time were about equal in both groups as seen in Table 1. In their respective groups an average of 6 traditional steel wires and 5 Flatwires were used to repair the sternum. Patients receiving the Flatwires reported significantly reduced pain at day of discharge compared to the steel wire cohort (3.07 vs. 4.92, p < 0.0066). Pain scores were statistically equal at 30 and 60 days (p < 0.149 and p < 0.088, respectively). Patients receiving the FlatWires did have shortened post-operative length of stay compared to those receiving steel wire; however, this difference was not found to be statistically significant (6.85 vs. 7.9 days, p < 0.093). Bypass pump off to surgery end time was used as an estimation of sternal closure time. Patients receiving the FlatWires had significantly increased closure time (74.39 vs. 56.39 minutes, p = 0.00025). Total cost during the patients’ hospital stay was reduced on average in the Flatwire cohort but this finding was not significant ($87,820.98 vs. $91,930.29, p < 0.3082). There were no perioperative complications attributed to FlatWire or standard steel wire. Chest X-ray at 30 days showed no sternal abnormalities in either group. Twenty-four of the 34 FlatWire patients were contacted by telephone at 1 year follow-up; there were no complications reported.

**Table 1:** Results

	**Figure 8 FlatWire**	**Standard wires**	**P value**
**Age**	64.1	64.7	0.409
**BMI**	30.42	29.43	0.238
**Days: surgery to discharge**	6.85	7.9	0.093
**VAS discharge**	3.07	4.92	0.0066
**VAS 30 days**	1.52	2.11	0.149
**VAS 60 days**	0.39	0.79	0.088
**Pump time**	110.16	101.04	0.212
**Time: Off pump to stop**	1:14:39	0:56:39	0.00025
**Cost: surgery to discharge**	87820.98	91930.29	0.3082

Statistical analysis of study results. (P < 0.05).

## Discussion

Complications following median sternotomy include infectious or noninfectious dehiscence, mediastinitis, osteomyelitis, chronic sternal pain and non-union. Comorbid patients often have poorer bone quality and impaired wound healing that make them more susceptible to these complications, particularly those with diabetes, osteoporosis, pulmonary disease or obesity. Physiologic loads sustained with coughing or valsalva may be sufficient to cause dehiscence in this high risk group [10]. The key factor is compromised sternal stability by wire breakage, excessive shearing forces, or lateral displacement of the sternal halves. The majority of dehiscence is caused by steel wires cutting through sternal bone. Some physicians select more durable closure methods rather than using the standard wire cerclage to avoid the unstable sternum [11]. Management of dehiscence varies by severity but often requires surgical debridement, rewiring, rigid plate fixation, or muscle flaps [12].

Post-sternotomy pain syndrome was first described in 1985 by Weber and colleagues, a condition most often caused by painful sternal wire sutures or protruding wires. It has been shown to be relieved in 83% of sufferers upon wire removal [13,14]. Other causes of post-sternotomy pain are sternal instability and, of course, cardiac ischemia. Prospective and retrospective cohort studies have indicated that the incidence of non-cardiac pain after sternotomy ranges from 7-28% [12,13,15]. Pain onset may be immediate or delayed and varies with comorbidities, closure technique and bone quality [11].

In our study, FlatWires significantly reduced pain after sternotomy compared to standard wire closure (p < 0.0066). A study by Wong et al., showed similar results in patients receiving rigid sternal fixation as opposed to conventional wire closure, as well as improved osteosynthesis at 3 and 6 months [16]. The findings are likely related to the superior mechanical properties of rigid fixation and the reduced cut into the bone. Decreased post-sternotomy pain may ultimately reduce analgesic requirements, patient dissatisfaction, and potential hospital visits. With less pain, patients may return to their normal level of activity sooner and avoid the detrimental sequelae of immobilization. They have improved quality of life and decreased incidence of anxiety and depression. Although rigid plate fixation is also more stable and associated with less pain than traditional steel wires, the cost is greater and the use of screws carries its own armory of complications (injury to mediastinal structures, screw migration) [11]. In addition, rigid fixation is contraindicated in patients with osteoporosis as good bone quality is required for screw placement.

Wire cerclage remains the standard among cardiovascular surgeons despite disadvantages due its familiarity, simplicity, and low cost [17]. Although the exact price of the FlatWire system has not yet been defined, it will be significantly less than current alternatives. This preliminary study showed the length of hospital stay was 6.8 days compared to 7.8 in the standard group; however this was not statistically significant. As the sample size is expanded, results may indicate an overall shorter hospital stay which would ultimately reduce hospital costs. Also, with a theoretical decrease in postoperative complications, hospital costs would be significantly reduced. A study of 201 patients in a Midwest hospital found that patients experiencing deep sternal infections stayed an additional 20 days in the hospital and incurred bills greater than $20,000 in those who survived [18]. Lower analgesic requirements secondary to reduced pain, earlier mobilization, or shorter recovery time may additionally provide financial advantages.

Time off pump to the surgery stop was increased in the FlatWire group compared to the control group by 15.9 minutes (p < 0.00025) on average. Although this was not a direct measurement of the time allotted for sternal closure it can be inferred that device application time was also longer. As with any new technology, there is a learning curve for physicians to become acquainted with a new device. It is reasonable to assume that the time for device application is expected to decrease as surgeons become more familiar with its use.

## Conclusion

This pilot study reports our initial experience using the Figure 8 FlatWire for sternal closure. Statistically significant decreases in post-operative pain and a potential reduction in length of hospital stay support the need for further research. The expanding prospective clinical trial aims to quantify sternal pain, patient risk factor stratification, operative duration, and postoperative complications in patients undergoing FlatWire closure in larger sample populations across other major institutions. Preliminary studies have shown FlatWires to provide superior biomechanical properties and less postoperative pain, characteristics known to reduce the incidence of post-sternotomy complications [7,8]. Further study of this new sternal closure device is warranted.

## Consent

Written informed consent was obtained from all patients for the publication of this report and any accompanying images.

## Abbreviations

BMI: Body mass index; CABG: Coronary artery bypass grafting.

## Competing interests

The authors declare that they have no competing interests.

## Authors’ contributions

KL, AB, and PG participated in developing the design of the study. PG performed the statistical analysis. AB and PG drafted the manuscript. All authors read and approved the final manuscript.
